# Eigenvalues of the covariance matrix as early warning signals for critical transitions in ecological systems

**DOI:** 10.1038/s41598-019-38961-5

**Published:** 2019-02-22

**Authors:** Shiyang Chen, Eamon B. O’Dea, John M. Drake, Bogdan I. Epureanu

**Affiliations:** 1 Department of Mechanical Engineering, University of Michigan, Ann Arbor, Michigan USA; 2 Odum School of Ecology, University of Georgia, Athens, Georgia USA; 3 Center for the Ecology of Infectious Diseases, University of Georgia, Athens, Georgia USA

## Abstract

Many ecological systems are subject critical transitions, which are abrupt changes to contrasting states triggered by small changes in some key component of the system. Temporal early warning signals such as the variance of a time series, and spatial early warning signals such as the spatial correlation in a snapshot of the system’s state, have been proposed to forecast critical transitions. However, temporal early warning signals do not take the spatial pattern into account, and past spatial indicators only examine one snapshot at a time. In this study, we propose the use of eigenvalues of the covariance matrix of multiple time series as early warning signals. We first show theoretically why these indicators may increase as the system moves closer to the critical transition. Then, we apply the method to simulated data from several spatial ecological models to demonstrate the method’s applicability. This method has the advantage that it takes into account only the fluctuations of the system about its equilibrium, thus eliminating the effects of any change in equilibrium values. The eigenvector associated with the largest eigenvalue of the covariance matrix is helpful for identifying the regions that are most vulnerable to the critical transition.

## Introduction

Ecological systems can be found in highly contrasting states. For example, shallow lakes may be either clear or turbid from an abundance of cyanobacteria^1^, coral reefs may be highly diverse or dominated by macroalgae^2^, areas of land can be either wooded, grassy and open^3^, or altogether barren^4^, and time series of climatic and biological indicators of oceans can contain abrupt jumps^5^. In fact, there is evidence^6–8^ that some transitions between all of these examples of contrasting states may occur abruptly as a consequence of a small change in one component of the system—for example, small changes in climate, ground water reduction, harvesting of certain species, and so on^9,10^. These easily triggered, abrupt changes among contrasting states are known as *critical transitions*.

A great deal of the support for the occurrence of critical transitions in nature relies on the linking of observations with mathematical models of the various ecological system. The critical transition corresponds to a movement of the mathematical state variables from one dynamical pattern to a qualitatively different one. As such, the critical transition is often modelled as a dynamic bifurcation. Such models not only represent how a drastic change in the system’s state may occur following a small change in some parameters, but also how this drastic change may be irreversible and unrelated to the behavior of the state variables prior to the bifurcation. Evidence of such irreversibility in ecological systems includes the observation that shallow lakes dominated by cyanobacteria do not return to a clear state immediately following the reduction of nutrient levels^11^. On the one hand, the irreversibility makes the transition more important to avert and therefore predict as a consequence of a given course of action. On the other hand, the abruptness of the transition rules out naive forecasting methods such as the projection of past trends in the state variables. Nevertheless, analysis of models has suggested a variety of methods that may indicate when a critical transition is imminent.

In many models that represent the critical transition as a movement from one stable equilibrium to another, the movement is preceded by decreasing asymptotic stability of the equilibrium from which the movement occurs. This mathematical phenomenon is referred to as *critical slowing down*^12^. The asymptotic stability of the equilibrium of a system of differential equations can often be determined by the eigenvalues of the Jacobian of the system evaluated at the equilibrium. The equilibrium is stable if the real part of all of the eigenvalues are negative and unstable if any real parts are positive. Critical slowing down occurs as one of the eigenvalues approaches zero and becomes less negative and thus the return to the equilibrium becomes slower. Thus in a completely deterministic, differential equation model, the rate at which small perturbations decay in the direction which the eigenvalue that is crossing zero pulls may form an indicator^13,14^. In models that include stochastic effects which interupt deterministic trajectories, other indicators have been proposed that estimate this return rate indirectly, such as the variance and autocorrelation of fluctuations in a time series of the observations of the state of a system. Such indicators of critical slowing down are not simply mathematical constructions; indicators calculated from observations of natural systems have been found to prefigure critical transitions as predicted by models^15–18^. Although the extent to which such indicators can be useful for predicting critical transitions in many practical situations remains unclear, progress in this area is being made on the theoretical front by the development of a rich variety of indicators, also known as early warning signals^19–26^.

For example, recent studies suggest that spatial patterns can also provide useful early warning signals^27–30^. In particular, Dakos *et al*.^28^ point out that an increase in spatial correlation of the state variables of spatial models can serve as an early warning signal of bifurcations. This is because, as the parameters of the model approach the bifurcation point, the state variables become slow in recovering from perturbations, which might lead to stronger fluctuations of state variables around their average values under a given intensity of random perturbations^31^. In such cases, the fluctuations of state variables around the spatial mean can also increase. The ability of fluctuations in one area to influence fluctuations in nearby areas then leads to larger correlations among neighboring units and, more generally, larger spatial correlations^32^. Therefore, spatial early warning signals, such as the spatial variance, spatial skewness and spatial correlation have been proposed as indicators for critical transitions of spatially extended systems. By referring to these measurements as “spatial” we simply mean that they are calculated from observations at different locations at a given time, a set of observations which we refer to as a snapshot. These spatial early warning signals have two major drawbacks. First, they are largely dependent on the deviation of state variables from their spatial mean. Thus, these indicators are affected by the change of the temporal mean of state variables at different locations as the system approaches a critical transition, which is especially likely to occur in heterogeneous systems in which parameters vary among locations. Additional comments on this issue are included in the Discussion section. Second, these methods only look at one snapshot at one time, thus limiting the information they can gather from the system. It is hard, for instance, to identify the temporal pattern that is associated with the critical transition using only a single snapshot.

This paper proposes a set of indicators based on the fluctuations of several state variables around their temporal average values under stochastic excitations (e.g., random environmental perturbations). The proposed indicators are based on the observation that for models with multiple state variables, the dynamics of certain sums of state variables become much slower than dynamics of other sums as the system approaches the critical transition. This is another example of critical slowing down. The sum of state variables which slows down is the vector projection of the state variables onto the dominant eigenvector of the Jacobian matrix of the model, evaluated at the model’s deterministic equilibrium. The rate at which this projected variable recovers from perturbations slows down as the bifurcation approaches because of the decreasing magnitude of the dominant eigenvalue. If the extent to which this projected variable is randomly perturbed is not commensurately decreased, its variance must increase. Thus, we propose the largest eigenvalue of the covariance matrix of the state variables and the percentage it accounts for of the total variation as multivariate early warning signals. One important assumption must be satisfied for the proposed early warning signals to work: namely, the critical transition may be modelled as a co-dimension one bifurcation. This assumption means that in a suitable model the bifurcation can be caused by the variation of a single quantity (i.e., a single parameter or a single correlated variation in several parameters). The proposed early warning signals can be applied to data from spatially heterogeneous systems (systems with different characteristics at different locations) if we define state variables as the state of the system at different locations.

Eigenvalues of the covariance matrix have been proposed in the past as an early warning signal for high dimensional systems^33,34^. It was established that the largest eigenvalue of the covariance matrix will increase close to a bifurcation. In our paper, we take a step further to show that for a system with a co-dimension one bifurcation, not only does the largest eigenvalue of the covariance matrix increase close to the bifurcation, it also becomes dominant compared to other eigenvalues of the covariance matrix. The contributions of this paper are threefold: (1) it examines a linear high dimensional stochastic system modeled by a Fokker-Planck equation and shows the relationship between the eigenvalues of the covariance matrix and the eigenvalues of the Jacobian matrix; (2) it shows how the eigenvalues of the covariance matrix and the percentage it accounts for of the total variation can be estimated and used as early warning signals for spatially correlated ecological systems when a more detailed model of the system is not available; and (3) it compares the proposed early warning signals with past spatial early warning signals and discusses advantages and drawbacks. It is important to note that early warning signals based on the critical slowing down phenomenon focus on bifurcations caused by the loss of linear stability. This also applies to the proposed early warning signals. For a nonlinear system, we assume the deviation from equilibrium is small and hence, a linear approximation is adequate. Researchers have proposed other early warning signals (based on basin size and so on) to anticipate critical transitions that may not be modelled as a loss of linear stability^35,36^.

## Results

### Main concept

Many natural and physical systems are high-dimensional, are constantly affected by random environmental perturbations, and can be modeled using first-order differential equations with noise terms^37–40^. Consider a nonlinear dynamical system with a vector **x**(*t*) of state variables described by first-order stochastic differential equations

1 $$d{\bf{x}}={\bf{f}}({\bf{x}})dt+{\bf{D}}({\bf{x}})d{\bf{W}}\mathrm{.}$$

The force vector **f**(**x**(*t*)) models the deterministic evolution of the system and we assume **f**(**x**(*t*)) does not depend on time *t* explicitly for simplicity. The deterministic system, *d***x**/*dt* = **f**(**x**) may have one or more equilibria which satisfy *d***x**/*dt* = **0**. We also assume that **f**(**x**(*t*)) is continuous and differentiable in the vicinity of an equilibrium of interest, which we denote ***μ***. *d***W** is a vector of Gaussian white noises with zero mean. The covariance matrix of the noise term is denoted by **D**(**x**).

Let **z**(*t*) = **x**(*t*)−***μ*** denote a vector of deviations from the equilibrium, where ***μ*** denotes the equilibrium of the state variables in the absence of noise. If the deviations are sufficiently small, little error occurs when we replace the force vector by its linear approximation

2 $${\bf{f}}({\bf{x}})\approx {\bf{f}}({\boldsymbol{\mu }})+{\bf{F}}{\bf{z}}={\bf{F}}{\bf{z}},$$

where **f**(***μ***) = 0 and **F** is the force matrix that determines the expected trajectory of **z** toward zero. In this study we assume **D**(**x**) to be independent of **x**. Due to the stochastic nature of the system, **z** is a random variable. Let *p*(**z**, *t*) be the probability density function that describes the likelihood of **z** falling within a particular range of values. The solution of the stochastic differential equations (Eq. 1) can be described using the probability density function *p*(**z**, *t*), which can be approximated as the solution to a linear Fokker-Planck equation^41^

3 $$\frac{\partial p({\bf{z}},t)}{\partial t}=\sum _{i,j\mathrm{=1}}^{N}-{F}_{ij}\frac{\partial ({z}_{j}p)}{\partial {z}_{i}}+\frac{1}{2}\sum _{i,j\mathrm{=1}}^{N}\,{D}_{ij}\frac{{\partial }^{2}p}{\partial {z}_{i}\partial {z}_{j}},$$

where **D** is the diffusion matrix that describes the covariance of a Gaussian white noise that acts on **z**. The initial condition of this Fokker-Planck equation is set to be *p*(**z**, *t*) = *δ*(**0**) and the boundary condition is *p*(±**∞**, *t*) = 0. The solution of this linear system is a multi-dimensional Gaussian density function^37^. We further consider that the fluctuations have reached a stationary distribution after the transients have died out. In such cases, the covariance matrix **Σ** of the solution, which describes the correlation between different state variables, is solely dependent on the force matrix **F** and the diffusion matrix **D**^41^. Therefore, we can use the covariance matrix **Σ**, which can be estimated directly from the measurements, to infer the characteristics of the force matrix **F** which is not necessarily available for a real dynamical system.

To calculate the covariance matrix **Σ**, we can use the decomposition of Kwon and coauthors^42^ which shows that **Σ** can be written as

4 $${\boldsymbol{\Sigma }}=-\,{{\bf{F}}}^{-1}({\bf{D}}+{\bf{Q}}\mathrm{)/2,}$$

where **Q** is an antisymmetric matrix with zeros on its diagonal which satisfies

5 $${\bf{F}}{\bf{Q}}+{\bf{Q}}{{\bf{F}}}^{\tau }={\bf{F}}{\bf{D}}-{\bf{D}}{{\bf{F}}}^{\tau },$$

where the superscript *τ* indicates the transpose.

Suppose **F** has distinct eigenvalues with negative real parts and that the dominant eigenvalue is real. Denote the eigenvalues of the covariance matrix **Σ** as *σ*_1_, *σ*_2_, …, *σ*_*N*_. In the Supplementary Material, we prove that under the assumption of a codimension one bifurcation, the largest eigenvalue of the covariance matrix *σ*_1_ becomes much larger than the other eigenvalues *σ*_2_, *σ*_3_, …, *σ*_*N*_ if the real part of the dominant eigenvalue of the force matrix becomes closer to 0 compared to the rest of the eigenvalues of the force matrix. This is because the dynamics along the direction of the eigenvector corresponding to the dominant eigenvalue become slower as the dominant eigenvalue of the Jacobian matrix approaches zero. Because the other eigenvalues are not approaching zero at the same rate as the dominant eigenvalue, the variance of the dynamics along that direction increases at a much higher rate. Thus, the largest eigenvalue of the covariance matrix and the percentage it accounts for of the total variation can be used as early warning signals.

The Fokker-Planck equation has been used in a number of papers to study a system approaching the bifurcation^43,44^. However, it is important to note that the solution to the linear Fokker-Planck equation (Eq. 3) is an approximation to the probability density function of the original system, which is the solution to a nonlinear Fokker-Planck equation that cannot be guaranteed to remain Gaussian as the system becomes arbitrarily close to the bifurcation point. In addition, at the bifurcation point, the probability density function of the original system will depend on time (even at long times), and the variance will grow over time. Nevertheless, we observed that the covariance of the Gaussian distribution obtained from the linearization is a good approximation close to the bifurcation point, as shown below, and can be used as an indicator of the system approaching bifurcation.

For the case where the dominant eigenvalues of **F** are complex conjugate pairs, the relationship between the largest eigenvalues of the covariance matrix and the real parts of the dominant eigenvalues are similar. The difference is that the subspace in which the variance of the dynamics increases at a much higher rate is now two-dimensional. For simplicity the example we give in the following sections only has real eigenvalues. Further comments about systems with complex conjugate pairs are also included in the Supplementary Material.

### Spatial ecological model

We consider a general 2D spatial model under the assumption that the space is discrete and the dynamics take place in an *n* × *n* square lattice which consists of coupled cells^45,46^. A 2D spatial model can be considered as a high dimensional system if we define state variables as the state of the system at different locations. The dynamics at each location (*i*, *j*) are affected by a reaction-type process, a diffusion process and a random excitation modeled by a random walk process *dW*_*i*,*j*_. The reaction-type process is described by the nonlinear deterministic function *g*_*i*,*j*_(*X*_*i*,*j*_, *r*(*i*, *j*), *c*). Each cell is also connected to its neighbors through a diffusive process. The general form of this model is

6 $$d{X}_{i,j}=({g}_{i,j}({X}_{i,j},r(i,j),\,c)+R({X}_{i+\mathrm{1,}j}+{X}_{i-\mathrm{1,}j}+{X}_{i,j+1}+{X}_{i,j-1}-4{X}_{i,j}))dt+\sigma d{W}_{i,j},$$

where *X*_*i*,*j*_ is the state variable at cell (*i*, *j*) in a 2D space, *r*_*i*,*j*_ is the heterogeneous parameter that changes with location, *c* is the bifurcation parameter, *R* is the constant dispersion rate, and *σ* is the standard deviation of the white noise *dW*_*i*,*j*_ at location (*i*, *j*). *i* = 1, 2, ..., *n* and *j* = 1, 2, ..., *n*. We use a symmetric boundary condition such that cells at the boundary have exchange only with neighboring cells and not with the boundary. Consequently, *X*_*n*+1,*l*_ = *X*_*n*,*l*_, *X*_*l*,*n*+1_ = *X*_*l*,*n*_, *X*_1,*l*_ = *X*_0,*l*_, and *X*_*l*,1_ = *X*_*l*,0_, for *l* = 1, 2, ..., *n*.

In the case that *σ* = 0, Eq. 6 is deterministic and has one or more equilibria which satisfy *dX*_*i*,*j*_/*dt* = 0. We use *μ*_*i*,*j*_ to denote the values of *μ*_*i*,*j*_ for one particular equilibrium of interest. In the neighborhood any *μ*_*i*,*j*_, the reaction-type process can be approximated by its linear approximation. Thus the probability density function of the state variables in Eq. 6 can be approximated by the Fokker-Planck Eq. 3 where **z** collects all the state variables in Eq. 6 concatenated and centered (i.e., *z*_*n*(*j*−1) +*i*_ = *X*_*i*,*j*_−*μ*_*i*,*j*_), **F** is set to the Jacobian matrix of the force function evaluated at the equilibrium, and **D** is the matrix describing the covariance of the random excitation. In this study, **D** is set for simplicity to be an identity matrix, which means noise terms at different cells are independent of each other. In the Supplementary Material we have shown that the choice of **D** does not affect the results as long as the stochastic perturbations from the environment do not concentrate on one patch.

The equation for the elements of the Jacobian matrix **F** can be expressed as

$${F}_{i,j}=(\begin{array}{ll}\frac{d{g}_{i}({z}_{i})}{d{z}_{i}}{|}_{{z}_{i}=0}-O(R), & {\rm{if}}\,i=j\\ R, & {\rm{if}}\,i-j=\mathrm{1,}\,i\,{\rm{mod}}\,n\ne 1\\ R, & {\rm{if}}\,j-i=\mathrm{1,}\,j\,{\rm{mod}}\,n\ne 1\\ R, & {\rm{if}}\,i-j=n\\ R, & {\rm{if}}\,j-i=n\\ 0, & {\rm{otherwise}}\end{array},$$

where *g*_*i*_ is the reaction function at cell ($[(i-\mathrm{1)}\,{\rm{mod}}\,n]+\mathrm{1,}\,\lceil i/n\rceil$), and *O*(*R*) is a term that is either 2*R*, 3*R*, or 4*R* depending on whether the cell has 2, 3, or 4 neighbors. Clearly **F** is a symmetric matrix containing real numbers. Thus, the eigenvalues of **F** must be real.

To illustrate how the proposed early warning signals can be used to anticipate critical transitions of an ecological system, we adapt three well-studied models^28,47,48^ to the form of Eq. 6 by adding the dispersion and noise terms. The first model describes the dynamics of biomass under harvesting. The second model describes the dynamics of nutrients of a eutrophic lake, and the third model describes the dynamics of macrophytes in a shallow lake. All three models have alternative stable states in their original form for some choice of parameters. Further, if a threshold value of the models’ parameters is crossed, one of the alternative stable states disappears and a rapid transition to the remaining stable equilibrium may occur. For example, the amount of biomass of the harvesting model is stable at an equilibrium with high population at a low harvesting rate. As the harvesting rate increases, the system collapses to a low population equilibrium. Details about the three models we use can be found in Table 1. The parameter values are obtained from Dakos and coauthors^28^. Heterogeneous parameters (*r*_*i*,*j*_) are introduced by randomly setting the value of *r*_*i*,*j*_ within a certain range. *n*^2^ = 20 × 20 = 400 cells are used in this model. The values of *r*_*i*,*j*_ we use in this study are included in the GitHub repository.

**Table 1:** Model details and parameter values used in the study.

Model and Parameter	Definition and value
Harvesting model	
$${g}_{i,j}({X}_{i,j},\,r(i,j),c)={r}_{i,j}{X}_{i,j}(1-\frac{{X}_{i,j}}{K})-c\frac{{X}_{i,j}^{2}}{{X}_{i,j}^{2}+1}$$	
*X*_*i*, *j*_	Resource biomass at cell (*i*, *j*); state variable
*c*	Maximum harvesting rate; bifurcation parameter
*r*_*i*, *j*_	Maximum growth rate at cell (*i*, *j*); heterogeneous parameter
*K*	Carrying capacity, 10
*R*	Dispersion rate, 0.2
*σ*	SD of white noise, 0.1
Eutrophication model	
$${g}_{i,j}({X}_{i,j},r(i,j),c)=a-{r}_{i,j}{X}_{i,j}+c\frac{{X}_{i,j}^{8}}{{X}_{i,j}^{8}+1}$$	
*X*_*i*, *j*_	Nutrient concentration at cell (*i*, *j*); state variable
*c*	Nutrient loading rate; bifurcation parameter
*r*_*i*, *j*_	Nutrient loss rate at cell (*i*, *j*); heterogeneous parameter
*a*	Maximum recycling rate, 0.5
*R*	Dispersion rate, 0.2
*σ*	SD of white noise, 0.05
Vegetation-turbidity model	
$${g}_{i,j}({X}_{i,j},\,r(i,\,j),\,c)={r}_{v}{X}_{i,j}(1-{X}_{i,j}\frac{{r}_{i,j}^{4}+{E}_{i,j}^{4}}{{r}_{i,j}^{4}})$$	
$${E}_{i,j}=\frac{{h}_{v}c}{{h}_{v}+{X}_{i,j}}$$	
*X*_*i*, *j*_	Vegetation cover at cell (*i*, *j*); state variable
*c*	Background turbidity; bifurcation parameter
*r*_*i*, *j*_	Half-saturation turbidity constant at cell (*i*, *j*); heterogeneous parameter
*r*_*v*_	Maximum vegetation growth rate, 0.5
*h*_*v*_	Half-saturation vegetation cover constant, 0.2
*R*	Dispersion rate, 0.2
*σ*	SD of white noise, 0.1

**Figure 1:**
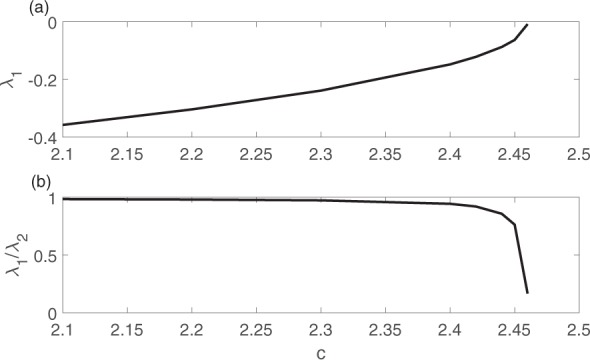
Variation of the eigenvalues of the spatial harvesting model; the largest eigenvalue (**a**) and the largest eigenvalue divided by the second largest eigenvalue (**b**) are shown versus the bifurcation parameter *c*.

The relationship between the dominant eigenvalue *λ*_1_ of the Jacobian matrix **J** and the bifurcation parameter *c* of the harvesting model is shown in Fig. 1a as an example. As the system moves toward the bifurcation, the dominant eigenvalue becomes closer to zero. Let *λ*_2_ be the second largest eigenvalue. The relationship between $\frac{{\lambda }_{1}}{{\lambda }_{2}}$ and the bifurcation parameter *c* is shown in Fig. 1b. When the system is far away from the bifurcation, *λ*_1_ is close to *λ*_2_. However, as the system moves toward the bifurcation, $\frac{{\lambda }_{1}}{{\lambda }_{2}}$ becomes closer to 0. Therefore, we expect to observe an increase of the largest eigenvalue of the covariance matrix as the system becomes closer to the bifurcation. The percentage the largest eigenvalue of the covariance matrix accounts for of the total variation is also expected to increase. The situation is similar for the other two models. To demonstrate how the proposed early warning signals can be used to forecast critical transitions, we apply the approach to all three spatial models in the following section.

### Early warning signals

**Figure 2:**
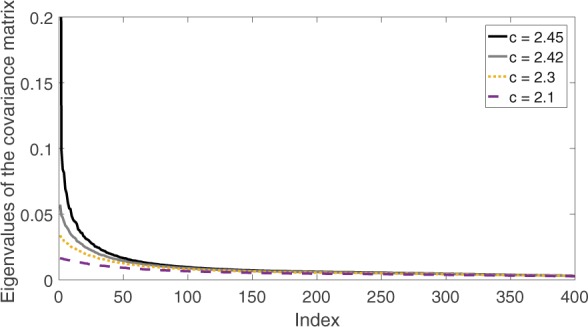
Change of the spectrum of the covariance matrix as the system moves toward the bifurcation at c = 2.47. The bifurcation point *c* = 2.47 is computed using the deterministic part of the harvesting model. Each line represents all the eigenvalues of the covariance matrix under a certain parameter value. The index is simply an integer which varies from 1 to 400, with index 1 meaning the largest eigenvalue, index 2 meaning the second largest eigenvalue, and so on.

We estimate the eigenvalues of the covariance matrix of the harvesting model at several different control parameter values as shown in Fig. 2. Each line shows all the eigenvalues of the covariance matrix in a descending order at a specific parameter value. The index is an integer which varies from 1 to 400, with index 1 meaning the largest eigenvalue, index 2 meaning the second largest eigenvalue, and so on.

As the system moves toward the bifurcation, the largest eigenvalue of the covariance matrix increases. Because the eigenvalues of the covariance matrix are identical to the variance of principal components, this discovery is consistent with the observation that the variance increases as the system moves toward the bifurcation. In particular, the largest eigenvalue of the covariance increases at a much larger rate than the other eigenvalues. Therefore, we propose two early warning signals:

- The first warning signal is the value of the largest eigenvalue of the covariance matrix *σ*_1_,
- The second warning signal is the percentage that the largest eigenvalue accounts for of the total variation $\frac{{\sigma }_{1}}{\sqrt{{\sigma }_{1}^{2}+\mathrm{...}+{\sigma }_{N}^{2}}}$, where *N* is the number of states.

**Figure 3:**
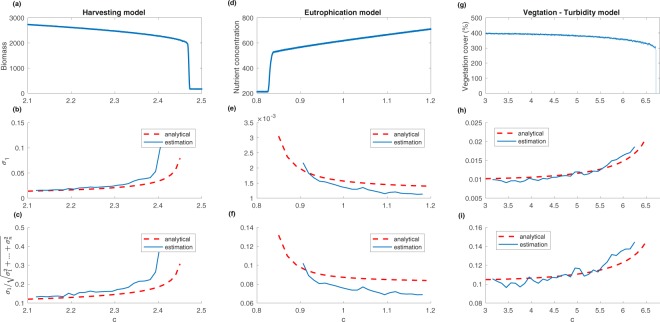
(**a,d,g**) Sum of the state variables as the bifurcation parameter *c* changes over time. (**b,e,h**) Largest eigenvalue *σ*_1_ of the covariance matrix estimated using a moving window as the bifurcation parameter *c* changes over time. (**c,f,i**) Largest eigenvalue of the covariance matrix over the Euclidean norm of a vector consisting of all the eigenvalues $\frac{{\sigma }_{1}}{\sqrt{{\sigma }_{1}^{2}+\mathrm{...}+{\sigma }_{n}^{2}}}$ estimated using a moving window as the bifurcation parameter *c* changes over time.

The relationship between the largest eigenvalue and the bifurcation parameter for all three models is shown in Fig. 3b,e,h. The eigenvalues are first calculated analytically using the decomposition described in the Main Concept. We also estimated the eigenvalues of the covariance matrix using a moving window on data obtained from a system with a time-varying bifurcation parameter *c*(*t*) = *c*_0_ + *δt*. In all simulations, time step *δ* is set to 1/16000. A total of 16000 snapshots are collected from the simulation. The moving window has a size of 1000 snapshots. As expected, the largest eigenvalue of the covariance matrix increases as the system moves toward the bifurcation point. Moreover, the largest eigenvalue of the covariance matrix becomes dominant as the dominant eigenvalue approaches 0. Therefore, Fig. 3c,f,i suggest that the percentage that the largest eigenvalue accounts for of the total variation also increases as the system approaches the bifurcation. The percentage as an early warning signal is particularly useful because it has a clear upper limit of 100%. That is in contrast to the largest eigenvalue of the covariance matrix, which theoretically can increase without bound.

It is important to note that the analytical expression of the covariance matrix is generally not available for a real system because the forms of the force matrix and the diffusion matrix are seldom known. However, we can estimate the covariance matrix directly from the time series data without relying on any prior knowledge of the system. Results from Fig. 3 show that the estimated eigenvalues of the covariance matrix agree quite well with the analytical eigenvalues close to the bifurcation. For parameters extremely close to the bifurcation point, the estimated eigenvalues generally have higher values compared to the analytical eigenvalues. This is caused by the moving window used in the estimation. The estimated eigenvalue is essentially an average of eigenvalues in that moving window. As the system approaches the bifurcation, the largest eigenvalue of the covariance matrix increases quickly, and that causes the early takeoff of the estimation compared to the analytical results. More details about the estimation method are presented in the Methods section, where we show that eigenvalues estimated using the shrinkage method agree well with the analytical results when a fixed parameter is used for simulation.

### Locations of tipping points

Another advantage of the proposed methods is that by examining the covariance matrix, we can obtain both the dominant eigenvalue and its corresponding eigenvector. We show in the Supplementary Material that as the dominant eigenvalue of the force matrix approaches zero, the dominant eigenvector of the covariance matrix approaches the dominant eigenvector of the force matrix. So by examining the eigenvector corresponding to the dominant eigenvalue, we can identify cells that are most affected by declining asymptotic stability of the equilibrium. Knowledge of these areas may provide crucial direction for monitoring and possibly preventing critical transitions.

To examine the relationship between the dominant eigenvector of the covariance matrix and vulnerable regions, we calculate local eigenvalues corresponding to groups of cells. By local eigenvalues we mean the dominant eigenvalue of the force matrix of a single cell or a local group of cells without considering the diffusion effects of the neighboring cells. Therefore, we assume that there is diffusion between cells within the local group, but not diffusion to cells that are outside the local group. We define a local group with size *i* × *i* at location (*j*, *k*) as cells:

7 $$[\begin{array}{cccc}(j,\,k) & (j,\,k+\mathrm{1)} & \cdots  & (j,\,k+i-\mathrm{1)}\\ (j+\mathrm{1,}\,k) & (j+\mathrm{1,}\,k+\mathrm{1)} & \cdots  & (j+\mathrm{1,}\,k+i-\mathrm{1)}\\ \vdots  & \vdots  & \ddots  & \vdots \\ (j+i-\mathrm{1,}\,k) & (j+i-\mathrm{1,}\,k+\mathrm{1)} & \cdots  & (j+i-\mathrm{1,}\,k+i-\mathrm{1)}\end{array}],$$

under the restriction that *j* + *i*−1, *k* + *i*−1 ≤ 20.

**Figure 4:**
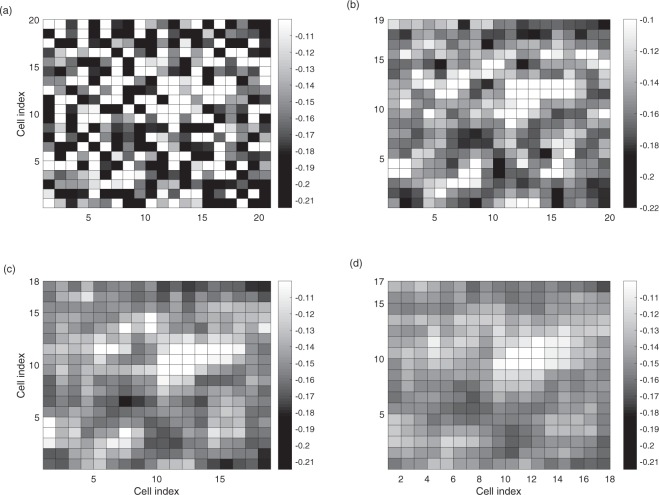
Local eigenvalues of the harvesting model. Only the dominant eigenvalues of the local areas are plotted. For each local group, the dominant eigenvalue is plotted at the upper left cell of the group. (**a**) 1 × 1, (**b**) 2 × 2, (**c**) 3 × 3 and (**d**) 4 × 4 cells are used to construct the local groups.

In Fig. 4, we show the dominant local eigenvalues of all the 1 × 1, 2 × 2, 3 × 3 and 4 × 4 local groups of the harvest model. Because of the diffusion effects, the dominant eigenvalues tend to decrease as the number of cells in the local group increases. The diffusion effect, on the other hand, does not have a significant effect on cells that are too far away from the local group. Therefore, the eigenvalue map stabilizes as the size of the local group reaches a certain level.

**Figure 5:**
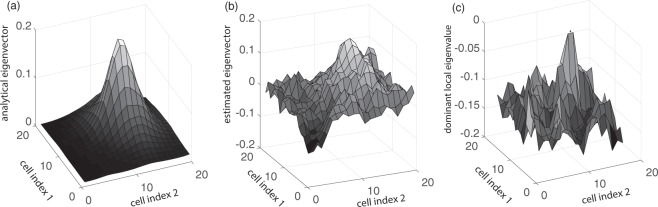
(**a**) The eigenvector corresponding to the dominant eigenvalue of the covariance matrix (analytical), (**b**) The eigenvector corresponding to the dominant eigenvalue of the covariance matrix (estimated using simulation data), (**c**) Dominant eigenvalues of the force matrix of the local cell groups. In (**a**,**b**), we transform the dominant eigenvector back to 2D form, and plot the amplitude for each state variable at the corresponding location.

Now we want to compare the dominant local eigenvalues of the harvesting model with its eigenvector corresponding to the dominant eigenvalue. Figure 5(a) exhibits the analytic eigenvector corresponding to the dominant eigenvalue of the covariance matrix, while Fig. 5(b) shows the eigenvector corresponding to the dominant eigenvalue estimated from simulation data. It is obvious that they share a dominant peak in the upper right region. There are other smaller peaks in Fig. 5(b) due to estimation errors. In Fig. 5(c), we show the dominant local eigenvalues of the 4 × 4 groups. It is obvious that the area with the largest amplitudes in the eigenvector corresponding to the dominant eigenvalue coincides with the area with the largest local eigenvalues. Thus, regions with local eigenvalues closest to 0 are identified as regions most vulnerable to critical transition by the eigenvector corresponding to the dominant eigenvalue.

## Discussion

We proposed two spatiotemporal early warning signals. These signals are based on the eigenvalues of the covariance matrix. Overall, our study suggests that an increase in the largest eigenvalue of the covariance matrix of a multivariate time series and the percentage it accounts for of the total variation can serve as early warning signals for critical transitions in multivariate systems. To provide a specific example, we have demonstrated an increase in these indicators as models of spatial ecological systems approach a bifurcation.

Here, we compared the two proposed early warning signals with past early warning signals^27^ including

1. spatial variance ${\sigma }^{2}=\frac{1}{HL}{\sum }_{h=1}^{H}\,{\sum }_{l=1}^{L}\,{({z}_{h,l}-\bar{{\bf{z}}})}^{2}$, where *H* and *L* are the number of cells in the vertical and horizontal direction, and $\bar{{\bf{z}}}$ is the mean value of **z** over the whole space.
2. spatial skewness $\gamma =\frac{1}{HL}{\sum }_{h=1}^{H}\,{\sum }_{l=1}^{L}\,\frac{{({z}_{h,l}-\bar{{\bf{z}}})}^{3}}{{\sigma }^{3}}$, where *σ* is the square root of the spatial variance.
3. spatial correlation function ${C}_{2}(r)=\frac{HL{\sum }_{i=1}^{H}\,{\sum }_{m=1}^{H}\,{\sum }_{j=1}^{L}\,{\sum }_{l=1}^{L}\,{w}_{i,j;m,l}({z}_{i,j}-\bar{{\bf{z}}})({z}_{m,l}-\bar{{\bf{z}}})}{W{\sum }_{l=1}^{L}\,{\sum }_{h=1}^{H}\,{({z}_{h,l}-\bar{{\bf{z}}})}^{2}}$, where *w*_*i*,*j*;*m*,*l*_ is 1 if spatial units [*i*, *j*] and [*m*, *l*] are separated by a distance *r*, and is 0 otherwise, and *W* is the total number of ordered pairs of units separated by the distance *r*.

The two proposed early warning signals are different from past ones in the sense that it does not take the variation of expected values of the state variables across space into account. Past spatial early warning signals are all affected by variation in the expected value of the state variables across space. To better explore this, we next take spatial variance as an example. The expected value of the spatial variance can be expressed as,

$$\begin{array}{rcl}{\rm{E}}({\hat{\sigma }}_{{\rm{spatial}}}^{2}) & = & {\rm{E}}(\frac{1}{M}\sum _{i=1}^{M}\,{({z}_{i}-\bar{{\bf{z}}})}^{2})\\  & = & \frac{M-1}{{M}^{2}}{\rm{E}}(\sum _{i=1}^{M}\,{z}_{i}^{2})-\frac{1}{{M}^{2}}\sum _{i\ne j}\,{\rm{E}}({z}_{i}{z}_{j})\\  & = & \frac{M-1}{{M}^{2}}\sum _{i=1}^{M}\,({\mu }_{i}^{2}+{{\rm{\Sigma }}}_{ii})-\frac{1}{{M}^{2}}\sum _{i\ne j}\,({\mu }_{i}{\mu }_{j}+{{\rm{\Sigma }}}_{ij}),\end{array}$$

where we used E(*z*_*i*_*z*_*j*_) = Σ_*ij*_ + *μ*_*i*_*μ*_*j*_. Thus we have

$$\begin{array}{rcl}{\rm{E}}({\hat{\sigma }}_{{\rm{spatial}}}^{2}) & = & \frac{1}{{M}^{2}}((M-1)\,\sum _{i=1}^{M}\,{\mu }_{i}^{2}-\sum _{i\ne j}\,{\mu }_{i}{\mu }_{j})\\  &  & +\,\frac{1}{{M}^{2}}((M-1)\,\sum _{i=1}^{M}\,{{\rm{\Sigma }}}_{ii}-\sum _{i\ne j}\,{{\rm{\Sigma }}}_{ij}),\end{array}$$

where ${\hat{\sigma }}_{{\rm{spatial}}}^{2}$ is the empirical spatial variance, *M* is the number of cells in the grid, *μ*_*i*_ is the expected value of state *i*, and **Σ** is the covariance matrix. Therefore, the spatial variance is affected by the expected values of the state variables (first half of the right hand side of the equation), and the covariance matrix (second half of the equation). As the parameters of the system change gradually, the expected value of the state variables may also vary. The changes in the indicators caused by the expected values, however, do not necessarily provide any information about the stability of the model’s equilibrium, especially for heterogeneous systems. Therefore, it is hard to predict in general how these proposed substitution spatial early warning signals are affected by the eigenvalues of the Jacobian of the model.

Next, we use an example to illustrate how the changes in mean values can affect the performance of spatial variance as an early warning signal. Simulation data is collected from a simple spatial model

8 $$d{X}_{i,j}=({r}_{i,j}c-{X}_{i,j}+R({X}_{i+\mathrm{1,}j}+{X}_{i-\mathrm{1,}j}+{X}_{i,j+1}+{X}_{i,j-1}-4{X}_{i,j}))dt+\sigma d{W}_{i,j},$$

where **r** is the same as the **r** we used for the harvesting model, and *c* is the control parameter. Other parameters are the same as in Table 1. In this simple model, there is no bifurcation as the control parameter *c* changes, therefore there is no critical slowing down.

**Figure 6:**
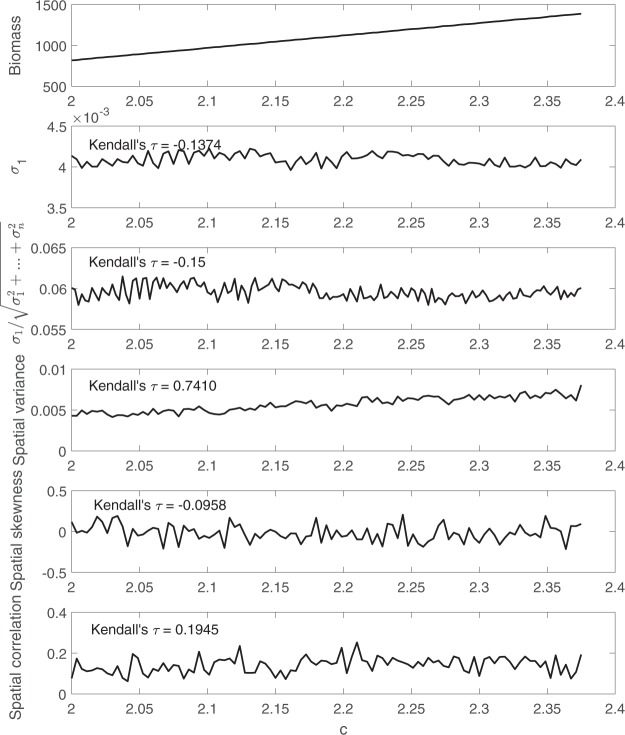
A comparison between the two proposed early warning signals, i.e. largest eigenvalue of the covariance matrix and the percentage it accounts for, with three past spatial early warning signals using simulation data obtained from system governed by 8.

Spatial early warning signals are calculated using simulation data, and are shown in Fig. 6. As the parameter *c* increases, values of the state variables also increase due to the change of their expected value. During this process, the largest eigenvalue of the covariance matrix remains stable due to a lack of critical slowing down. Spatial variance, on the contrary, has a clear upward trend. To compare different spatial early warning signals, we quantified their trend using the nonparametric Kendall’s *τ* rank correlation of the control parameter *c* and the spatial early warning signals. In this example, the spatial variance is strongly affected by the change of expected values with a Kendall’s *τ* of over 0.74.

**Figure 7:**
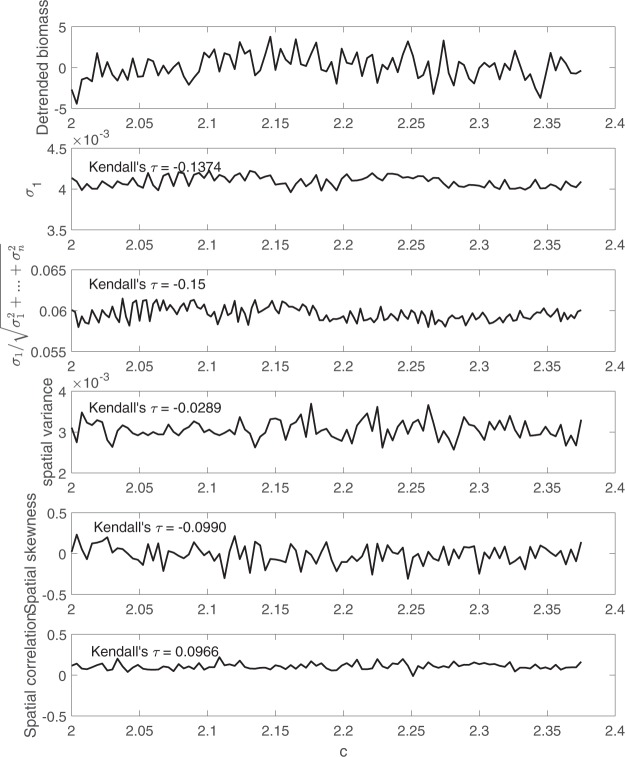
The comparison between the two proposed early warning signals, i.e. largest eigenvalue of the covariance matrix and the percentage it accounts for, with three past spatial early warning signals using detrended simulation data obtained from Model 8.

To better illustrate this point, all the spatial early warning signals are calculated again using detrended simulation data as shown in Fig. 7. As expected, the upward trend in spatial variance has disappeared after detrending the simulation data. Therefore, it is highly recommended to detrend the data before applying such spatial early warning signals.

**Figure 8:**
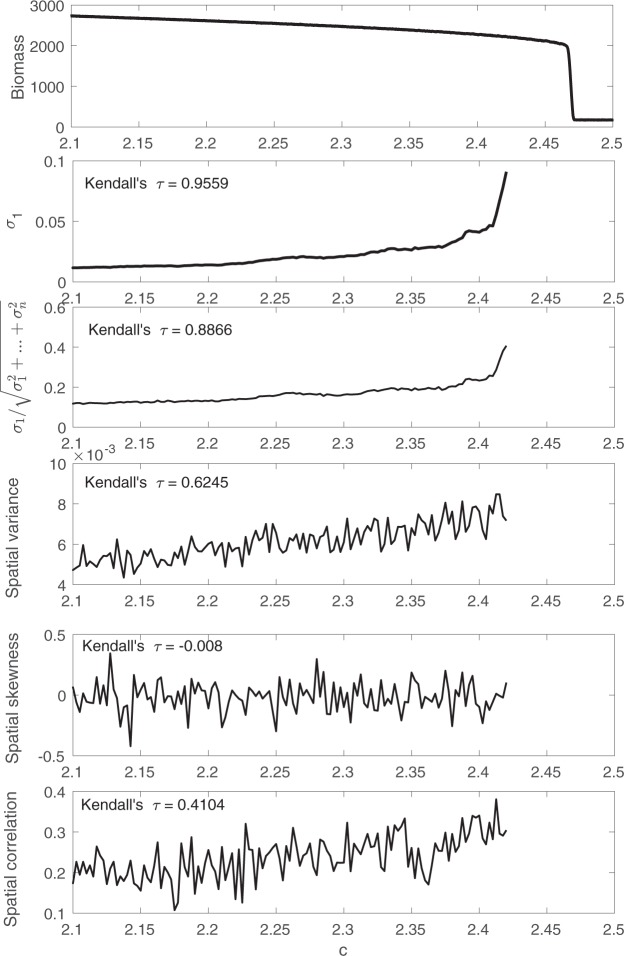
A comparison between the two proposed early warning signals, i.e. largest eigenvalue of the covariance matrix and the percentage it accounts for, with three past spatial early warning signals using detrended simulation data obtained from the harvesting model.

Next, we apply the proposed and past early warning signals to the detrended simulation data from the harvesting model. The results are shown in Fig. 8.

As the system moves towards the bifurcation, both the spatial variance and the spatial correlation increase, while the spatial skewness does not have an obvious trend. The proposed early warning signals, i.e. the largest eigenvalue of the covariance matrix and the ratio ${\sigma }_{1}/\sqrt{{\sigma }_{1}^{2}+\mathrm{...}+{\sigma }_{n}^{2}}$, have the largest Kendall’s *τ*, which indicates that they have the most consistent upward trend compared to other spatial early warning signals. Moreover, the proposed early warning signals have sharp increases close to the bifurcation, while others increase linearly. Therefore, the sharp increase in the proposed early warning signals can be seen as an indication that the system is approaching the bifurcation.

In conclusion, this study explored how eigenvalues and eigenvectors of a spatial covariance matrix related to critical transitions in spatially extended ecological systems.

Eigenvalues of the covariance matrix better capture critical slowing down due to their direct relationship with the eigenvalues of the force matrix that characterizes the dynamics, compared to past spatial early warning signals. We therefore propose to use the largest eigenvalue of the covariance matrix as a spatial early warning signal. By establishing the relationship between the eigenvalues of the covariance matrix and the eigenvalues of the force matrix, we mathematically show that the dominance of the largest eigenvalue of the covariance matrix can also be used as an early warning signal. We compared these proposed signals with proposed substitution ones. The proposed early warning signals can potentially be applied to other high dimensional systems, such as multispecies systems^33^, complex structures^49^ and so on.

This approach may be used to identify also the vulnerable regions in a spatially correlated system. By studying the eigenvector corresponding to the dominant eigenvalue of the covariance matrix (the vector which determines the coordinates of the first principal component), we can obtain important clues regarding the region where the transition is most likely to occur. The correlation between the vulnerable regions and this eigenvector of the covariance matrix will become stronger as the dominant eigenvalue of the Jacobian approaches zero. This means that the percentage of the total variation explained by the dominant eigenvalue of the covariance matrix is an indicator not only of a potential critical transition, but also of a better opportunity for dimension reduction.

## Methods

### Covariance matrix estimation

Here we show the process of covariance matrix estimation by choosing a parameter value close to the bifurcation, simulating the model to obtain a stationary response to the random excitation, and estimating the covariance matrix.

**Figure 9:**
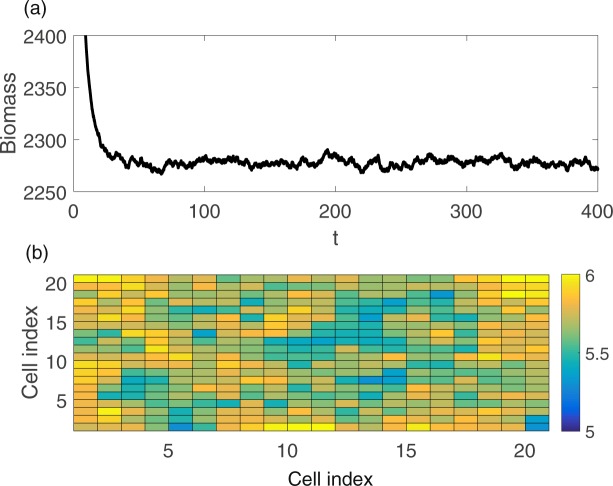
(**a**) An example of the time series of the total amount of biomass when the bifurcation parameter *c* is 2.4. (**b**) A snapshot of the state variable values at each cell.

An example of simulation results is shown in Fig. 9a, where the sum of the biomass is plotted. Three different ways are used to estimate the covariance matrix, i.e.:

1. Unbiased empirical covariance matrix.
2. Shrinkage approach.
3. Analytical method.

First, the unbiased empirical covariance matrix **S** is used to estimate the covariance matrix. Each entry of **S** is defined as

9 $${S}_{jk}=\frac{1}{n-1}\sum _{i=1}^{n}\,({x}_{ij}-{\bar{x}}_{j})\,({x}_{ik}-{\bar{x}}_{k})$$

where *x*_*ij*_ is the *i*_th_ measurement collected at the *j*_th_ cell, and ${\bar{x}}_{j}$ is the average of measurements collected at the *j*_th_ cell over time, *n* is the number of snapshots used for the estimation.

However, the unbiased empirical covariance matrix is not a good estimate of the covariance matrix when the number of snapshots is small compared to the number of variables, as pointed out in^50,51^. This is because the sample covariance matrix **S** might not be positive definite anymore when only a small number of snapshots are available. In such cases, the sample covariance matrix tends to overestimate the value of its largest eigenvalues, while underestimate the rest. A shrinkage approach can be used to estimate the covariance matrix under such circumstances. The linear shrinkage approach suggests to combine a high-dimensional unconstrained estimate **S** and a low-dimensional constrained estimate **T** in a weighted average given by

10 $${{\bf{S}}}^{\ast }=\eta {\bf{S}}+\mathrm{(1}-\eta ){\bf{T}},$$

where S^*^ is the regularized estimate, 1−*η* ∈ [0, 1] is the shrinkage intensity. *η* is estimated by minimizing a squared error loss risk function which is a combination of mean square error and variance as shown in^50^. The low-dimensional constrained estimate **T** is chosen based on the presumed lower-dimensional structure in the data set. In the case of the spatial harvesting model, the assumption is that the variance along different directions does not decrease dramatically after the first few principal components. Examples of values of **T** can be found in Schäfer and coauthors^50^.

**Figure 10:**
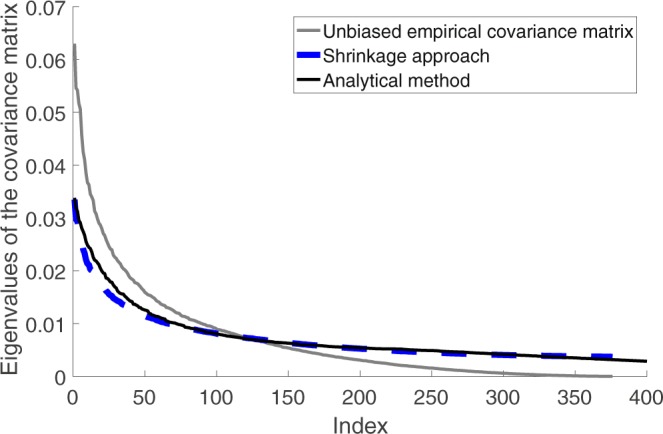
Estimation of eigenvalues of the covariance matrix using three different methods: analytical covariance matrix, sample covariance matrix, shrinkage estimation method. *c* = 2.4 is used to obtain the simulation data. 200 snapshot are used for the covariance matrix estimation.

The covariance matrix is obtained analytically also by calculating the Jacobian matrix **F** of the deterministic solution and using the decomposition method described above. The analytical covariance matrix is used as a benchmark for the estimation results. Eigenvalues estimated by the three methods are calculated and plotted in Fig. 10. The R package *corpcor* is used for the covariance matrix estimation. In particular, the function *cov.shrink* is used for the shrinkage estimations. Default parameters are used. As expected, the shrinkage method outperforms the sample covariance matrix in estimating the eigenvalues.

## Supplementary information

Supplementary Information

## Data Availability

All necessary codes for simulations and analysis will be made available at Github following publication.

## References

[1] Scheffer M, Rinaldi S, Gragnani A, Mur LR, Nes EHV (1997). On the Dominance of Filamentous Cyanobacteria in Shallow. Turbid Lakes. Ecol..

[2] Knowlton N (1992). Thresholds and Multiple Stable States in Coral Reef Community Dynamics. Integr Comp Biol.

[3] Dublin HT, Sinclair A, McGlade J (1990). Elephants and Fire as Causes of Multiple Stable States in the Serengeti-Mara Woodlands. J Anim Ecol.

[4] van de Koppel J, Rietkerk M, Weissing FJ (1997). Catastrophic vegetation shifts and soil degradation in terrestrial grazing systems. Trends Ecol. & Evol..

[5] Hare SR, Mantua NJ (2000). Empirical evidence for North Pacific regime shifts in 1977 and 1989. Prog. Ocean..

[6] Scheffer M, Carpenter S, Foley JA, Folke C, Walker B (2001). Catastrophic shifts in ecosystems. Nat..

[7] Scheffer M, Carpenter SR (2003). Catastrophic regime shifts in ecosystems: linking theory to observation. Trends Ecol. & Evol..

[8] Rietkerk M, Dekker SC, de Ruiter PC, van de Koppel J (2004). Self-organized patchiness and catastrophic shifts in ecosystems. Sci..

[9] Tilman D (2001). Forecasting agriculturally driven global environmental change. Sci..

[10] Vitousek PM, Mooney HA, Lubchenco J, Melillo JM (1997). Human domination of earth’s ecosystems. Sci..

[11] Meijer, M. L. Biomanipulation in the Netherlands : 15 years of experience. *Ph.D. thesis, S.n., S.l*., http://library.wur.nl/WebQuery/wurpubs/65524 (2000).

[12] Scheffer M (2009). Early-warning signals for critical transitions. Nat..

[13] Wissel C (1984). A universal law of the characteristic return time near thresholds. Oecologia.

[14] Van Nes EH, Scheffer M (2007). Slow recovery from perturbations as a generic indicator of a nearby catastrophic shift. The Am. Nat..

[15] Drake JM, Griffen BD (2010). Early warning signals of extinction in deteriorating environments. Nat..

[16] Lim J, Epureanu BI (2011). Forecasting a class of bifurcations: Theory and experiment. Phys. Rev. E.

[17] Veraart AJ (2012). Recovery rates reflect distance to a tipping point in a living system. Nat..

[18] Dai L, Vorselen D, Korolev KS, Gore J (2012). Generic Indicators for Loss of Resilience Before a Tipping Point Leading to Population Collapse. Sci..

[19] Dakos V (2012). Methods for detecting early warnings of critical transitions in time series illustrated using simulated ecological data. PloS One.

[20] O’Regan SM, Drake JM (2013). Theory of early warning signals of disease emergence and leading indicators of elimination. Theor. Ecol..

[21] Ghadami A, Cesnik CE, Epureanu BI (2018). Model-less forecasting of Hopf bifurcations in fluid-structural systems. J. Fluids Struct..

[22] D’Souza K, Epureanu BI, Pascual M (2015). Forecasting bifurcations from large perturbation recoveries in feedback ecosystems. PloS One.

[23] Chen, L., Liu, R., Liu, Z.-P., Li, M. & Aihara, K. Detecting early-warning signals for sudden deterioration of complex diseases by dynamical network biomarkers. *Sci. Reports* **2** (2012). 10.1038/srep00342 PMC3314989 22461973

[24] Squartini, T., Van Lelyveld, I. & Garlaschelli, D. Early-warning signals of topological collapse in interbank networks. *Sci. Reports* **3** (2013). 10.1038/srep03357 PMC3842548 24285089

[25] Chen S, Epureanu B (2018). Forecasting bifurcations in parametrically excited systems. Nonlinear Dyn..

[26] O’Dea, E. B., Park, A. W. & Drake, J. M. Estimating the distance to an epidemic threshold. *J. Roy. Soc. Interface* **15**, 20180034 (2018). 10.1098/rsif.2018.0034 PMC6030631 29950512

[27] K´efi S (2014). Early warning signals of ecological transitions: Methods for spatial patterns. PloS One.

[28] Dakos V, van Nes EH, Donangelo R, Fort H, Scheffer M (2010). Spatial correlation as leading indicator of catastrophic shifts. Theor. Ecol..

[29] Guttal V, Jayaprakash C (2009). Spatial variance and spatial skewness: leading indicators of regime shifts in spatial ecological systems. Theor. Ecol..

[30] Dai L, Korolev KS, Gore J (2013). Slower recovery in space before collapse of connected populations. Nat..

[31] Ives AR (1995). Measuring resilience in stochastic systems. Ecol. Monogr..

[32] Dakos V, K´efi S, Rietkerk M, Van Nes EH, Scheffer M (2011). Slowing down in spatially patterned ecosystems at the brink of collapse. The Am. Nat..

[33] Dakos, V. Identifying best-indicator species for abrupt transitions in multispecies communities. *Ecol. Indic* (2017).

[34] Brock, W. & Carpenter, S. Variance as a leading indicator of regime shift in ecosystem services. *Ecol. Soc*. **11** (2006).

[35] Menck PJ, Heitzig J, Marwan N, Kurths J (2013). How basin stability complements the linear-stability paradigm. Nat. physics.

[36] Nolting BC, Abbott KC (2016). Balls, cups, and quasi-potentials: quantifying stability in stochastic systems. Ecol..

[37] Van Kampen, N. G. *Stochastic Processes in Physics and Chemistry*, vol. 1 (Elsevier, 1992).

[38] Wilkinson DJ (2009). Stochastic modelling for quantitative description of heterogeneous biological systems. Nat. Rev. Genet..

[39] Gammaitoni L (1995). Stochastic resonance and the dithering effect in threshold physical systems. Phys. Rev. E.

[40] Neubert MG, Caswell H (1997). Alternatives to resilience for measuring the responses of ecological systems to perturbations. Ecol..

[41] Risken, H. Fokker-planck equation. In The Fokker-Planck Equation, 63–95 (Springer, 1996).

[42] Kwon C, Ao P, Thouless DJ (2005). Structure of stochastic dynamics near fixed points. Proc. Natl. Acad. Sci. United States Am..

[43] Knobloch E, Wiesenfeld K (1983). Bifurcations in fluctuating systems: The center-manifold approach. J. Stat. Phys..

[44] Kuehn C (2011). A mathematical framework for critical transitions: Bifurcations, fast–slow systems and stochastic dynamics. Phys. D: Nonlinear Phenom..

[45] Keitt TH, Lewis MA, Holt RD (2001). Allee effects, invasion pinning, and species’ borders. The Am. Nat..

[46] van Nes EH, Scheffer M (2005). Implications of spatial heterogeneity for catastrophic regime shifts in ecosystems. Ecol..

[47] Carpenter SR, Ludwig D, Brock WA (1999). Management of eutrophication for lakes subject to potentially irreversible change. Ecol. applications.

[48] Scheffer, M. The story of some shallow lakes. In *Ecology of shallow lakes*, 1–19 (Springer, 2004).

[49] Sohn H, Czarnecki JA, Farrar CR (2000). Structural health monitoring using statistical process control. J. Struct. Eng..

[50] Sch¨afer J (2005). A shrinkage approach to large-scale covariance matrix estimation and implications for functional genomics. Stat. Appl. Genet. Mol. Biol..

[51] Efron, B. Maximum likelihood and decision theory. *The Annals Stat*. 340–356 (1982).

