# Supplementary material for: Eigenvalues of the covariance matrix as early warning signals for critical transitions in ecological systems
Source: Sci Rep. 2019 Feb 22;9:2572. doi: 10.1038/s41598-019-38961-5 (PMC6385210; doi:10.1038/s41598-019-38961-5)
Supplement: Supplementary file 1 — Supplementary Information [file 41598_2019_38961_MOESM1_ESM.pdf]

Supplementary Materials: Eigenvalues of the covariance matrix as early warning signals for critical transitions in ecological systems

Shiyang Chen,[1] Eamon O'Dea,[2,3] John M. Drake,[2,3] and Bogdan Epureanu[1]
[1]*Department of Mechanical Engineering, University of Michigan, Ann Arbor, Michigan, USA*
[2]*Odum School of Ecology, University of Georgia, Athens, Georgia, USA*
[3]*Center for the Ecology of Infectious Diseases, University of Georgia, Athens, Georgia, USA*
(Dated: June 21, 2018)

We begin by stating our main assumptions and notation in the next section. Following that, our main analytic results are all stated without proof for the sake of conciseness. Proofs are provided in the final section, with the results restated for convenience.

I. ASSUMPTIONS AND NOTATION

Consider a system of N state variables $z_1, \ldots, z_N$ that have a probability density $p(\mathbf{z}, t)$ that satisfies the linear Fokker-Planck equation

$$\frac{\partial p(\mathbf{z}, t)}{\partial t} = \sum_{i,j=1}^{N} -F_{ij} \frac{\partial (z_j p)}{\partial z_i} + \frac{1}{2} \sum_{i,j=1}^{N} D_{ij} \frac{\partial^2 p}{\partial z_i \partial z_j}$$

with a force matrix $\mathbf{F}$ and a diffusion matrix $\mathbf{D}$. Assume that all of the eigenvalues of $\mathbf{F}$ are distinct with negative real parts and are denoted $\lambda_1, \lambda_2, \ldots, \lambda_N$. These eigenvalues are indexed from smallest to largest in terms of the value of their real part (i.e., $|\mathrm{Re}(\lambda_1)| \leq \ldots \leq |\mathrm{Re}(\lambda_N)|$). The diffusion matrix $\mathbf{D}$ is assumed to have all positive eigenvalues. With these assumptions, the stationary distribution of $\mathbf{z}$ is Gaussian and we denote its covariance matrix as $\mathbf{\Sigma}$. Our results concern the relationship between the eigenvalues of $\mathbf{F}$ and $\mathbf{\Sigma}$ as the system undergoes a codimension-1 bifurcation. In such a bifurcation, typically only one of $\mathbf{F}$'s eigenvalues—or the real part of one complex conjugate pair of $\mathbf{F}$'s eigenvalues—vanishes at the critical transition.

II. EIGENVALUES OF THE COVARIANCE MATRIX

Lemma 1. *Let the columns of a matrix* $\mathbf{T}$ *contain the eigenvectors of* $\mathbf{F}$*. Let* $\tilde{\mathbf{\Sigma}}$ *be the covariance of the state variables if the eigenvectors are used as their coordinate basis. That is,* $\tilde{\mathbf{\Sigma}} = \mathbf{T}^{-1}\mathbf{\Sigma}\mathbf{T}^{-\tau}$*. Then the elements of* $\tilde{\mathbf{\Sigma}}$ *satisfy*

$$\tilde{\Sigma}_{ij} = -\frac{\tilde{D}_{ij}}{\lambda_i + \lambda_j}$$

where $\tilde{\mathbf{D}} = \mathbf{T}^{-1}\mathbf{D}\mathbf{T}^{-\tau}$*.*

Lemma 2. *Suppose that the dominant eigenvalue of* $\mathbf{F}$ *is real and also suppose that* $|\tilde{D}_{11}/(2\lambda_1)|\epsilon \geq |\max(\tilde{\mathbf{D}})/(\lambda_2)|$ *with* $0 < \epsilon \ll 1$*. Then the dominant eigenvalue of* $\mathbf{\Sigma}$ *is equal to* $|\tilde{D}_{11}/(2\lambda_1)| + O(\epsilon|\tilde{\Sigma}_{11}|)$ *and all of the other eigenvalues are* $O(\epsilon|\tilde{\Sigma}_{11}|)$*.*

Lemma 3. *Suppose that the dominant eigenvalues of* $\mathbf{F}$ *form a complex conjugate pair. Further suppose that* $|\tilde{D}_{12}/(2\mathrm{Re}(\lambda_1))|\epsilon \geq |\max(\tilde{\mathbf{D}})/\lambda_3|$*, with* $0 < \epsilon \ll 1$*. Then the sum of the two largest eigenvalues of* $\mathbf{\Sigma}$ *is* $O(|\tilde{D}_{12}/\mathrm{Re}(\lambda_1)|)$ *and all of the other eigenvalues are* $O(\epsilon|\tilde{\Sigma}_{12}|)$*.*

Theorem 4. *Suppose that the magnitude of the real part of a dominant eigenvalue of* $\mathbf{F}$ *is small such that the assumptions of either Lemmas 2 or 3 are satisfied. Then as this real part approaches zero, if the assumptions of Lemma 2 are satisfied, the largest eigenvalue of* $\mathbf{\Sigma}$ *becomes larger absolutely and larger relative to all of the other eigenvalues of* $\mathbf{\Sigma}$*. Alternatively, if the assumptions of Lemma 3 are satisfied, the sum of the largest two eigenvalues of* $\mathbf{\Sigma}$ *becomes larger absolutely and relative to all of the other eigenvalues of* $\mathbf{\Sigma}$*.*

III. PROOFS

Lemma 1. *Let the columns of a matrix* $\mathbf{T}$ *contain the eigenvectors of* $\mathbf{F}$. *Let* $\tilde{\mathbf{\Sigma}}$ *be the covariance of the state variables if the eigenvectors are used as their coordinate basis. That is,* $\tilde{\mathbf{\Sigma}} = \mathbf{T}^{-1}\mathbf{\Sigma}\mathbf{T}^{-\tau}$. *Then the elements of* $\tilde{\mathbf{\Sigma}}$ *satisfy*

$$\tilde{\Sigma}_{ij} = -\frac{\tilde{D}_{ij}}{\lambda_i + \lambda_j} \tag{1}$$

where $\tilde{\mathbf{D}} = \mathbf{T}^{-1}\mathbf{D}\mathbf{T}^{-\tau}$.

Proof. Kwon and coauthors[2] show that the covariance matrix $\mathbf{\Sigma}$ may be written as

$$\mathbf{\Sigma} = -\mathbf{F}^{-1}(\mathbf{D}+\mathbf{Q})/2, \tag{2}$$

where $\mathbf{Q}$ is an antisymmetric matrix with zeroes on its diagonal which satisfies

$$\mathbf{F}\mathbf{Q} + \mathbf{Q}\mathbf{F}^{\tau} = \mathbf{F}\mathbf{D} - \mathbf{D}\mathbf{F}^{\tau}. \tag{3}$$

Next, let $\tilde{\mathbf{Q}} = \mathbf{T}^{-1}\mathbf{Q}\mathbf{T}^{-\tau}$ and $\mathbf{\Lambda} = \mathbf{T}^{-1}\mathbf{F}\mathbf{T}$, the diagonalization of $\mathbf{F}$. Equation (3) repeated in terms of these matrices is

$$\mathbf{\Lambda}\tilde{\mathbf{Q}} + \tilde{\mathbf{Q}}\mathbf{\Lambda} = \mathbf{\Lambda}\tilde{\mathbf{D}} - \tilde{\mathbf{D}}\mathbf{\Lambda}. \tag{4}$$

Thus the elements of $\tilde{\mathbf{Q}}$ must satisfy

$$(\lambda_i + \lambda_j)\tilde{Q}_{ij} = (\lambda_i - \lambda_j)\tilde{D}_{ij}. \tag{5}$$

To use (5) to find elements of $\tilde{\Sigma}$, note that (2) holds in transformed coordinates as

$$\tilde{\mathbf{\Sigma}} = -\mathbf{\Lambda}^{-1}(\tilde{\mathbf{D}}+\tilde{\mathbf{Q}})/2. \tag{6}$$

Putting (5) and (6) together yields (1). □

Lemma 2. *Suppose that the dominant eigenvalue of* $\mathbf{F}$ *is real and also suppose that* $|\tilde{D}_{11}/(2\lambda_1)|\epsilon \geq |\max(\tilde{\mathbf{D}})/(\lambda_2)|$ *with* $0 < \epsilon \ll 1$. *Then the dominant eigenvalue of* $\mathbf{\Sigma}$ *is equal to* $|\tilde{D}_{11}/(2\lambda_1)| + O(\epsilon|\tilde{\Sigma}_{11}|)$ *and all of the other eigenvalues are* $O(\epsilon|\tilde{\Sigma}_{11}|)$.

Proof. By hypothesis, all of the elements of $\tilde{\mathbf{\Sigma}}$ besides $\tilde{\Sigma}_{11}$ are at least a factor of $1/\epsilon$ smaller in magnitude than $\tilde{\mathbf{\Sigma}}_{11}$. We denote this difference in magnitude by noting that $\tilde{\Sigma}_{ij} = O(\epsilon|\tilde{\Sigma}_{11}|)$ for $(i,j) \neq (1,1)$. With this notation, we can write $\tilde{\mathbf{\Sigma}}$ as

$$\tilde{\mathbf{\Sigma}} = \begin{pmatrix} -\frac{\tilde{D}_{11}}{2\lambda_1} & O(\epsilon|\tilde{\Sigma}_{11}|) & \cdots & O(\epsilon|\tilde{\Sigma}_{11}|) \\ O(\epsilon|\tilde{\Sigma}_{11}|) & O(\epsilon|\tilde{\Sigma}_{11}|) & \cdots & O(\epsilon|\tilde{\Sigma}_{11}|) \\ \vdots & \vdots & \ddots & \vdots \\ O(\epsilon|\tilde{\Sigma}_{11}|) & O(\epsilon|\tilde{\Sigma}_{11}|) & \cdots & O(\epsilon|\tilde{\Sigma}_{11}|) \end{pmatrix}. \tag{7}$$

Along with $\mathbf{\Sigma} = \mathbf{T}\tilde{\mathbf{\Sigma}}\mathbf{T}^{\tau}$, (7) yields

$$\mathbf{\Sigma} = \mathbf{T}_1 \left|\frac{\tilde{D}_{11}}{2\lambda_1}\right| \mathbf{T}_1^{\tau} + \begin{pmatrix} O(\epsilon|\tilde{\Sigma}_{11}|) & \cdots & O(\epsilon|\tilde{\Sigma}_{11}|) \\ \vdots & \ddots & \vdots \\ O(\epsilon|\tilde{\Sigma}_{11}|) & \cdots & O(\epsilon|\tilde{\Sigma}_{11}|) \end{pmatrix}. \tag{8}$$

Postmultiplying both sides of (8) by $\mathbf{T}_1$ yields

$$\mathbf{\Sigma}\mathbf{T}_1 = \left|\frac{\tilde{D}_{11}}{2\lambda_1}\right| \mathbf{T}_1 + \begin{pmatrix} O(\epsilon|\tilde{\Sigma}_{11}|) \\ \vdots \\ O(\epsilon|\tilde{\Sigma}_{11}|) \end{pmatrix}. \tag{9}$$

Clearly, $|\tilde{D}_{11}/(2\lambda_1)| + O(\epsilon|\tilde{\Sigma}_{11}|)$ is an eigenvalue of $\mathbf{\Sigma}$. Because $\mathbf{\Sigma}$ is a covariance matrix, all of its eigenvalues are non-negative. These eigenvalues must sum to the trace of $\mathbf{\Sigma}$, which is $|\tilde{D}_{11}/(2\lambda_1)| + O(\epsilon|\tilde{\Sigma}_{11}|)$ according to (8). Hence all of the other eigenvalues must be $O(\epsilon|\tilde{\Sigma}_{11}|)$. □

Lemma 3. *Suppose that the dominant eigenvalues of* $\mathbf{F}$ *form a complex conjugate pair. Further suppose that* $|\tilde{D}_{12}/(2\mathrm{Re}(\lambda_1))|\epsilon \geq |\max(\tilde{\mathbf{D}})/\lambda_3|$*, with* $0 < \epsilon \ll 1$*. Then the sum of the two largest eigenvalues of* $\mathbf{\Sigma}$ *is* $O(|\tilde{D}_{12}/\mathrm{Re}(\lambda_1)|)$ *and all of the other eigenvalues are* $O(\epsilon|\tilde{\Sigma}_{12}|)$*.*

Proof. Our proof uses the same general approach to the case where λ_1 is real. Using (1), we can write $\tilde{\mathbf{\Sigma}}$ as

$$\tilde{\mathbf{\Sigma}} = \begin{pmatrix} -\frac{\tilde{D}_{11}}{2\lambda_1} & -\frac{\tilde{D}_{12}}{2\mathrm{Re}(\lambda_1)} & O(\epsilon|\tilde{\Sigma}_{12}|) & \cdots & O(\epsilon|\tilde{\Sigma}_{12}|) \\ -\frac{\tilde{D}_{12}}{2\mathrm{Re}(\lambda_1)} & -\frac{\tilde{D}_{11}}{2\lambda_1} & O(\epsilon|\tilde{\Sigma}_{12}|) & \cdots & O(\epsilon|\tilde{\Sigma}_{12}|) \\ O(\epsilon|\tilde{\Sigma}_{12}|) & O(\epsilon|\tilde{\Sigma}_{12}|) & O(\epsilon|\tilde{\Sigma}_{12}|) & \cdots & O(\epsilon|\tilde{\Sigma}_{12}|) \\ \vdots & \vdots & \vdots & \ddots & \vdots \\ O(\epsilon|\tilde{\Sigma}_{12}|) & O(\epsilon|\tilde{\Sigma}_{12}|) & O(\epsilon|\tilde{\Sigma}_{12}|) & \cdots & O(\epsilon|\tilde{\Sigma}_{12}|) \end{pmatrix} \tag{10}$$

which along with $\mathbf{\Sigma} = \mathbf{T}\tilde{\mathbf{\Sigma}}\mathbf{T}^\tau$ yields

$$\mathbf{\Sigma} = (\mathbf{T}_1, \mathbf{T}_2) \begin{pmatrix} -\frac{\tilde{D}_{11}}{2\lambda_1} & -\frac{\tilde{D}_{12}}{2\mathrm{Re}(\lambda_1)} \\ -\frac{\tilde{D}_{12}}{2\mathrm{Re}(\lambda_1)} & -\frac{\tilde{D}_{11}}{2\lambda_1} \end{pmatrix} (\mathbf{T}_1, \mathbf{T}_2)^\tau + \begin{pmatrix} O(\epsilon|\tilde{\Sigma}_{12}|) & \cdots & O(\epsilon|\tilde{\Sigma}_{12}|) \\ \vdots & \ddots & \vdots \\ O(\epsilon|\tilde{\Sigma}_{12}|) & \cdots & O(\epsilon|\tilde{\Sigma}_{12}|) \end{pmatrix}. \tag{11}$$

Alternatively $\mathbf{\Sigma}$ may be factorized as

$$\mathbf{\Sigma} = (\mathrm{Re}(\mathbf{T}_1), \mathrm{Im}(\mathbf{T}_1))\mathbf{\Sigma}^{(1)}(\mathrm{Re}(\mathbf{T}_1), \mathrm{Im}(\mathbf{T}_1))^\tau + \begin{pmatrix} O(\epsilon|\tilde{\Sigma}_{12}|) & \cdots & O(\epsilon|\tilde{\Sigma}_{12}|) \\ \vdots & \ddots & \vdots \\ O(\epsilon|\tilde{\Sigma}_{12}|) & \cdots & O(\epsilon|\tilde{\Sigma}_{12}|) \end{pmatrix}, \tag{12}$$

where $\mathbf{\Sigma}^{(1)}$ is a real 2×2 matrix that satisfies

$$\mathbf{\Sigma}^{(1)} = \mathbf{T}^{(1)}\tilde{\mathbf{\Sigma}}^{(1)}(\mathbf{T}^{(1)})^\tau \tag{13}$$

where

$$\tilde{\mathbf{\Sigma}}^{(1)} = \begin{pmatrix} -\frac{\tilde{D}_{11}}{2\lambda_1} & -\frac{\tilde{D}_{12}}{2\mathrm{Re}(\lambda_1)} \\ -\frac{\tilde{D}_{12}}{2\mathrm{Re}(\lambda_1)} & -\frac{\tilde{D}_{11}}{2\lambda_1} \end{pmatrix} \tag{14}$$

and $\mathbf{T}^{(1)}$ is a matrix whose columns are eigenvectors of

$$\mathbf{F}^{(1)} = \begin{pmatrix} \mathrm{Re}(\lambda_1) & \mathrm{Im}(\lambda_1) \\ -\mathrm{Im}(\lambda_1) & \mathrm{Re}(\lambda_1) \end{pmatrix}. \tag{15}$$

Specifically,

$$\mathbf{T}^{(1)} = \begin{pmatrix} 1 & 1 \\ i & -i \end{pmatrix} \tag{16}$$

so that

$$(\mathbf{T}_1, \mathbf{T}_2) = (\mathrm{Re}(\mathbf{T}_1), \mathrm{Im}(\mathbf{T}_1))\mathbf{T}^{(1)}. \tag{17}$$

The matrix $\mathbf{\Sigma}^{(1)}$ represents the covariance in a two-dimensional subspace spanned by $\mathrm{Re}(\mathbf{T}_1)$ and $\mathrm{Im}(\mathbf{T}_1)$. Now let $\mathbf{H}$ be

$$\begin{pmatrix} |\frac{\tilde{D}_{11}}{2\lambda_1}|^2 + |\frac{\tilde{D}_{12}}{2\mathrm{Re}(\lambda_1)}|^2 & \frac{\tilde{D}_{11}\tilde{D}_{12}}{4\lambda_1\mathrm{Re}(\lambda_1)} + \overline{\frac{\tilde{D}_{11}}{\lambda_1}}\frac{\tilde{D}_{12}}{4\mathrm{Re}(\lambda_1)} \\ \frac{\tilde{D}_{11}\tilde{D}_{12}}{4\lambda_1\mathrm{Re}(\lambda_1)} + \overline{\frac{\tilde{D}_{11}}{\lambda_1}}\frac{\tilde{D}_{12}}{4\mathrm{Re}(\lambda_1)} & |\frac{\tilde{D}_{11}}{2\lambda_1}|^2 + |\frac{\tilde{D}_{12}}{2\mathrm{Re}(\lambda_1)}|^2 \end{pmatrix}, \tag{18}$$

the product of $\tilde{\mathbf{\Sigma}}^{(1)}$ with its conjugate transpose. It is straightforward to verify that the eigenvalues of $\mathbf{H}$ are

$$h_1^2 = \left(\frac{|\tilde{D}_{11}|}{2|\lambda_1|} + \frac{|\tilde{D}_{12}|}{2\ \mathrm{Re}(\lambda_1)}\right)^2, \tag{19}$$

$$h_2^2 = \left(\frac{|\tilde{D}_{11}|}{2|\lambda_1|} - \frac{|\tilde{D}_{12}|}{2\ \mathrm{Re}(\lambda_1)}\right)^2. \tag{20}$$

If $|\tilde{D}_{11}| > 0$, the associated eigenvectors are the columns of

$$\mathbf{A} = \begin{pmatrix} \frac{\tilde{D}_{11}\tilde{D}_{12}}{2\lambda_1 \,\mathrm{Re}(\lambda_1)n} & \frac{\tilde{D}_{11}\tilde{D}_{12}}{2\lambda_1 \,\mathrm{Re}(\lambda_1)n} \\ \frac{|\tilde{D}_{11}|\tilde{D}_{12}}{2|\lambda_1| \,\mathrm{Re}(\lambda_1)n} & -\frac{|\tilde{D}_{11}|\tilde{D}_{12}}{2|\lambda_1| \,\mathrm{Re}(\lambda_1)n} \end{pmatrix}, \tag{21}$$

where the normalizing constant n ensures that the eigenvectors have unit norms. The Takagi factorization[1] of $\tilde{\mathbf{\Sigma}}^{(1)}$ is then

$$\tilde{\mathbf{\Sigma}}^{(1)} = \mathbf{AP} \,\mathrm{diag}(h_1, h_2)\, \mathbf{P}^{\tau}\mathbf{A}^{\tau} \tag{22}$$

where h_1 and h_2 are positive and $\mathbf{P}$ is a diagonal matrix of phase factors that satisfies

$$\mathbf{P}^2 = \bar{\mathbf{A}}^{\tau}\tilde{\mathbf{\Sigma}}^{(1)}\bar{\mathbf{A}} \,\mathrm{diag}(1/h_1, 1/h_2). \tag{23}$$

If $|\tilde{D}_{11}| = 0$, then $\tilde{\mathbf{\Sigma}}^{(1)}$ is a real symmetric matrix. It can be decomposed in the form of (22) by letting

$$\mathbf{A} = 1/\sqrt{2}\begin{pmatrix} -1 & -1 \\ 1 & -1 \end{pmatrix} \tag{24}$$

and

$$\mathbf{P} = \begin{pmatrix} i & 0 \\ 0 & 1 \end{pmatrix}. \tag{25}$$

Now let $\mathbf{M} = (\mathrm{Re}(\mathbf{T}_1), \mathrm{Im}(\mathbf{T}_1))\mathbf{T}^{(1)}\mathbf{AP} \,\mathrm{diag}(\sqrt{h_1}, \sqrt{h_2})$. Note from (12), (13), and (22) that

$$\mathbf{\Sigma} = \mathbf{MM}^{\tau} + \begin{pmatrix} O(\epsilon|\tilde{\Sigma}_{12}|) & \cdots & O(\epsilon|\tilde{\Sigma}_{12}|) \\ \vdots & \ddots & \vdots \\ O(\epsilon|\tilde{\Sigma}_{12}|) & \cdots & O(\epsilon|\tilde{\Sigma}_{12}|) \end{pmatrix}. \tag{26}$$

To obtain a simple equation for the eigenvalues of $\mathbf{\Sigma}$, we next decompose $\mathbf{M}$ into the product of an orthonormal matrix $\mathbf{V}$ and an upper triangular matrix $\mathbf{U}$. This can be done by applying the Gram-Shmidt process. The resulting first column of $\mathbf{V}$ is simply $\mathbf{M}_1/|\mathbf{M}_1|$ where $\mathbf{M}_1$ is the first column of $\mathbf{M}$. Let $\tilde{\mathbf{V}}_2 = \mathbf{M}_2 - \frac{\mathbf{M}_1^{\tau}\mathbf{M}_2}{\mathbf{M}_1^{\tau}\mathbf{M}_1}\mathbf{M}_1$. The second column of $\mathbf{V}$ is $\tilde{\mathbf{V}}_2/|\tilde{\mathbf{V}}_2|$. The matrix $\mathbf{U}$ is thus

$$\begin{pmatrix} |\mathbf{M}_1| & |\mathbf{M}_1|\frac{\mathbf{M}_2^{\tau}\mathbf{M}_1}{\mathbf{M}_1^{\tau}\mathbf{M}_1} \\ 0 & |\tilde{\mathbf{V}}_2| \end{pmatrix}. \tag{27}$$

Now, since columns of $\mathbf{V}$ form an orthonormal basis, the eigenvalues of $\mathbf{MM}^{\tau} = \mathbf{VUU}^{\tau}\mathbf{V}^{\tau}$ are identical to those of $\mathbf{UU}^{\tau}$. From (26), we can approximate to within $O(\epsilon|\tilde{\Sigma}_{12}|)$ two eigenvalues of $\mathbf{\Sigma}$ as those of $\mathbf{MM}^{\tau}$. The trace of $\mathbf{UU}^{\tau}$ turns out to be equal to

$$\begin{cases} h_1 + h_2 + (h_1 - h_2)\frac{\mathrm{Re}(\bar{\lambda}_1\tilde{D}_{11})}{|\tilde{D}_{11}||\lambda_1|}\,\mathrm{Re}(\mathbf{T}_1^{\tau}\mathbf{T}_1) + (h_2 - h_1)\frac{\mathrm{Im}(\bar{\lambda}_1\tilde{D}_{11})}{|\tilde{D}_{11}||\lambda_1|}\,\mathrm{Im}(\mathbf{T}_1^{\tau}\mathbf{T}_1), & \text{for } |\tilde{D}_{11}| > 0 \\ h_1 + h_2, & \text{otherwise.} \end{cases} \tag{28}$$

To further simplify this expression, we note that $|\tilde{\Sigma}_{11}| \leq |\tilde{\Sigma}_{12}|$. To see this, note that from (13) and (16) it follows that $|\tilde{\Sigma}_{11}|^2 - |\tilde{\Sigma}_{12}|^2 = [(\Sigma_{12}^{(1)})^2 - \Sigma_{11}^{(1)}\Sigma_{22}^{(1)}]/4 \leq 0$. Then from (19) and (20) we can conclude that $h_1 + h_2 = 2|\tilde{\Sigma}_{12}|$ and $h_1 - h_2 = -2|\tilde{\Sigma}_{11}|$. Thus the trace of $\mathbf{UU}^{\tau}$ may also be written as

$$\begin{cases} \left|\frac{\tilde{D}_{12}}{\mathrm{Re}(\lambda_1)}\right| + \left|\frac{\tilde{D}_{11}}{\lambda_1}\right|\left(\frac{\mathrm{Im}(\bar{\lambda}_1\tilde{D}_{11})}{|\tilde{D}_{11}||\lambda_1|}\,\mathrm{Im}(\mathbf{T}_1^{\tau}\mathbf{T}_1) - \frac{\mathrm{Re}(\bar{\lambda}_1\tilde{D}_{11})}{|\tilde{D}_{11}||\lambda_1|}\,\mathrm{Re}(\mathbf{T}_1^{\tau}\mathbf{T}_1)\right), & \text{for } |\tilde{D}_{11}| > 0 \\ |\frac{\tilde{D}_{12}}{\mathrm{Re}(\lambda_1)}|, & \text{otherwise.} \end{cases} \tag{29}$$

This expression is always $O(|\tilde{D}_{12}/\mathrm{Re}(\lambda_1)|)$ because $|\tilde{D}_{12}/(\mathrm{Re}(\lambda_1))|$ is the largest term: It follows from $|\tilde{\Sigma}_{11}| \leq |\tilde{\Sigma}_{12}|$ that $|\tilde{D}_{12}/\,\mathrm{Re}(\lambda_1)| \geq |\tilde{D}_{11}/\lambda_1|$ and the coefficient of $|\tilde{D}_{11}/\lambda_1|$ is the difference of two terms that are less than one. From (26) and the fact that the eigenvectors of $\mathbf{\Sigma}$ must be orthogonal, all of the other eigenvalues of $\mathbf{\Sigma}$ must be $O(\epsilon|\tilde{\Sigma}_{12}|)$. □

Theorem 4. *Suppose that the magnitude of the real part of a dominant eigenvalue of* $\mathbf{F}$ *is small such that the assumptions of either Lemmas 2 or 3 are satisfied. Then as this real part approaches zero, if the assumptions of Lemma 2 are satisfied, the largest eigenvalue of* $\mathbf{\Sigma}$ *becomes larger absolutely and larger relative to all of the other eigenvalues of* $\mathbf{\Sigma}$*. Alternatively, if the assumptions of Lemma 3 are satisfied, the sum of the largest two eigenvalues of* $\mathbf{\Sigma}$ *becomes larger absolutely and relative to all of the other eigenvalues of* $\mathbf{\Sigma}$*.*

Theorem 4 follows straightforwardly from Lemmas 2 and 3.

---

[1] Roger A Horn and Charles R Johnson. *Matrix analysis.* Cambridge university press, 2012.
[2] Chulan Kwon, Ping Ao, and David J Thouless. Structure of stochastic dynamics near fixed points. *Proceedings of the National Academy of Sciences of the United States of America*, 102(37):13029–13033, 2005.
